# Supplementary material for: Relationships between community composition, productivity and invasion resistance in semi-natural bacterial microcosms
Source: eLife. 2021 Oct 18;10:e71811. doi: 10.7554/eLife.71811 (PMC8523168; doi:10.7554/eLife.71811)
Supplement: Supplementary file 1. [file elife-71811-supp1.docx]

**Supplementary File 1:** Functional group membership – taxonomic assignments for each of the OTUs in each of the 18 functional groups identified using the functionInk approach

| **Cluster/functional group** | **OTUs (Nearest species-level designation)** |
| --- | --- |
| **1** | 1. *Arthrobacter protophormiae* 2. *Arthrobacter sulfureus* 3. *Arthrobacter.sp.* 4. *Arthrobacter.spp.* |
| **2** | 1. *Comamonas spp.* 2. *Pseudomonas lutea* 3. *Pseudomonas marincola* 4. *Pseudomonas migulae* 5. *Pseudomonas plecoglossicida* 6. *Pseudomonas putida* 7. *Pseudomonas rhizosphaerae* 8. *Pseudomonas syringae* 9. *Pseudomonas thivervalensis* 10. *Pseudomonas tolaasii* 11. *Pseudomonas trivialis* 12. *Pseudomonas veronii* 13. *Pseudomonas vranovensis* 14. *Stenotrophomonas rhizophila* |
| **3** | 1. *Bacillus thermoamylovorans* 2. *Cloacibacterium spp.* 3. *Corynebacterium diphtheriae* |
| **5** | 1. *Acinetobacter genomospecies 3* 2. *Acinetobacter towneri* 3. *Novispirillum itersonii* 4. *Ralstonia pickettii* |
| **8** | 1. *Chryseobacterium gregarium* 2. *Dysgonomonas spp.* 3. *Pedobacter aquatilis* 4. *Pedobacter spp.* |
| **13** | 1. *Hylemonella spp.* 2. *Acidovorax konjaci* 3. *Variovorax spp.* |
| **14** | 1. *Streptomyces sanglieri* 2. *Streptomyces xanthochromogenes* 3. *Streptomyces viridochromogenes* |
| **16** | 1. *Janthinobacterium lividum* 2. *Novosphingobium subarcticum* 3. *Pedobacter daejeonensis* |
| **17** | 1. *Acinetobacter piperi* 2. *Massilia spp.* 3. *Pedobacter cryoconitis* |
| **18** | 1. *Cedecea spp.* 2. *Citrobacter werkmanii* 3. *Erwinia persicina* 4. *Erwinia rhapontici* 5. *Escherichia shigella spp.* 6. *Klebsiella pneumoniae* 7. *Pantoea agglomerans* 8. *Pantoea vagens* 9. *Serratia fonticola* 10. *Serratia liquefaciens* 11. *Serratia quinivorans* 12. *Trabulsiella spp.* |
| **20** | 1. *Acidovorax spp.* 2. *Acinetobacter calcoaceticus* 3. *Acinetobacter johnsonii* 4. *Aquabacterium spp.* 5. *Brevundimonas aurantiaca* 6. *Caenimonas spp.* 7. *Delftia lacustris* 8. *Herbaspirillum rubrisubalbicans* 9. *Herbaspirillum spp.* 10. *Leptothrix spp.* 11. *Massilia timonae* 12. *Paucimonas spp.* 13. *Phenylobacterium spp.* 14. *Pseudomonas balearica* 15. *Pseudomonas pseudoalcaligenes* 16. *Stenotrophomonas maltophilia* |
| **22** | 1. *Chryseobacterium soldanellicola* 2. *Epilithonimonas lactis* 3. *Pedobacter wanjuense* |
| **23** | 1. *Rhizobium cellulosilyticus* 2. *Rhizobium giardinii* 3. *Rhizobium leguminosarum* |
| **24** | 1. *Haemophilus parainfluenzae* 2. *Neisseria subflava* 3. *Streptococcus salivarius* |
| **27** | 1. *Bosea thiooxidans* 2. *Brevundimonas bullata* 3. *Brevundimonas variabilis* |
| **28** | 1. *Corynebacteriales spp.* 2. *Nocardioides spp.* 3. *Pleomorphomonas spp.* 4. *Vogesella spp.* |
| **35** | 1. *Pseudoxanthomonas taiwanensis* 2. *Tepidimonas spp.* 3. *Weeksella spp.* |
| **39** | 1. *Agrobacterium tumefaciens* 2. *Rhizobium spp.* 3. *Rhizobium sullae* |
| **45 (combined with clusters 48, 49, 56)** | 1. *Paenibacillus borealis* 2. *Paenibacillus chondroitinus* 3. *Paenibacillus favisporus* 4. *Paenibacillus lautus* 5. *Paenibacillus wynnii* 6. *Paenibacillus xylanilyticus* 7. *Paenibacillus.spp.* |
