# Supplementary material for: Relationships between community composition, productivity and invasion resistance in semi-natural bacterial microcosms
Source: eLife. 2021 Oct 18;10:e71811. doi: 10.7554/eLife.71811 (PMC8523168; doi:10.7554/eLife.71811)
Supplement: Transparent reporting form [file elife-71811-transrepform.docx]

***eLife’s* transparent reporting form**

We encourage authors to provide detailed information *within their submission* to facilitate the interpretation and replication of experiments. Authors can upload supporting documentation to indicate the use of appropriate reporting guidelines for health-related research (see [EQUATOR Network](about:blank)), life science research (see the [BioSharing Information Resource](about:blank)), or the [ARRIVE guidelines](about:blank) for reporting work involving animal research. Where applicable, authors should refer to any relevant reporting standards documents in this form.

If you have any questions, please consult our Journal Policies and/or contact us: [editorial@elifesciences.org](about:blank).

**Sample-size estimation**

- You should state whether an appropriate sample size was computed when the study was being designed
- You should state the statistical method of sample size computation and any required assumptions
- If no explicit power analysis was used, you should describe how you decided what sample (replicate) size (number) to use

We do not think this applies to our submission as we did explicitly not estimate sample size during the study design; we were limited to the size cryopreserved archive of hundreds of microbial communities (after accounting for missing data etc.) for these experiments. However, this was an unusually large sample size compared to similar experiments in the field.

Please outline where this information can be found within the submission (e.g., sections or figure legends), or explain why this information doesn’t apply to your submission:

**Replicates**

- You should report how often each experiment was performed
- You should include a definition of biological versus technical replication
- The data obtained should be provided and sufficient information should be provided to indicate the number of independent biological and/or technical replicates
- If you encountered any outliers, you should describe how these were handled
- Criteria for exclusion/inclusion of data should be clearly stated
- High-throughput sequence data should be uploaded before submission, with a private link for reviewers provided (these are available from both GEO and ArrayExpress)

Each phenotypic assay was performed 4 times (pseudoreplicates/technical replicates) to generate the mean value across assays, which was used for the analysis. The biological replicates are therefore communities, the technical replicates are therefore the 4 assays performed on each community. This information can be found in Methods: Laboratory techniques: Invader survival.

Please outline where this information can be found within the submission (e.g., sections or figure legends), or explain why this information doesn’t apply to your submission:

**Statistical reporting**

- Statistical analysis methods should be described and justified
- Raw data should be presented in figures whenever informative to do so (typically when N per group is less than 10)
- For each experiment, you should identify the statistical tests used, exact values of N, definitions of center, methods of multiple test correction, and dispersion and precision measures (e.g., mean, median, SD, SEM, confidence intervals; and, for the major substantive results, a measure of effect size (e.g., Pearson's r, Cohen's d)
- Report exact p-values wherever possible alongside the summary statistics and 95% confidence intervals. These should be reported for all key questions and not only when the p-value is less than 0.05.

Please outline where this information can be found within the submission (e.g., sections or figure legends), or explain why this information doesn’t apply to your submission:

We used random forests and structural equation modelling for this study, reporting appropriate metrics for each within the Results section of the MS and within Figures 2 and 4 where feasible. The random forest regressions and structural equation models are all reproducible via our provided code, and can also be downloaded directly from our Open Science Framework data repository (<https://doi.org/10.17605/OSF.IO/HC57W> - see 3_end). We also include linear fits to some of the data in Figure 3 including R^2^ values – this is not a formal part of the analysis, but facilitates quick comparison of trends with previous studies (e.g. diversity-invasion relationships).

(For large datasets, or papers with a very large number of statistical tests, you may upload a single

table file with tests, Ns, etc., with reference to sections in the manuscript.)

**Group allocation**

- Indicate how samples were allocated into experimental groups (in the case of clinical studies, please specify allocation to treatment method); if randomization was used, please also state if restricted randomization was applied
- Indicate if masking was used during group allocation, data collection and/or data analysis

Not applicable – no experimental groups.

Please outline where this information can be found within the submission (e.g., sections or figure legends), or explain why this information doesn’t apply to your submission:

**Additional data files (“source data”)**

- We encourage you to upload relevant additional data files, such as numerical data that are represented as a graph in a figure, or as a summary table
- Where provided, these should be in the most useful format, and they can be uploaded as “Source data” files linked to a main figure or table
- Include model definition files including the full list of parameters used
- Include code used for data analysis (e.g., R, MatLab)
- Avoid stating that data files are “available upon request”

Please indicate the figures or tables for which source data files have been provided:

Code for this analysis freely is available at [https://github.com/befriendabacterium/communityinvasion](about:blank)

Data for the analysis at raw, pre-analysis and end-result stages is available at <https://doi.org/10.17605/OSF.IO/HC57W>.
